# Changes in well-being among socially isolated older people during the COVID-19 pandemic: An outcome-wide analysis

**DOI:** 10.1073/pnas.2308697121

**Published:** 2024-04-22

**Authors:** Claryn S. J. Kung, Andrew Steptoe

**Affiliations:** ^a^Department of Behavioural Science and Health, University College London, London WC1E 6BT, United Kingdom

**Keywords:** mental health, loneliness, life satisfaction, health behaviors, internet use

## Abstract

Older adults experienced major changes during the COVID-19 pandemic and ensuing restrictions, and it might be expected that those who were already socially isolated before the pandemic were particularly vulnerable. We apply an outcome-wide longitudinal design on 4,636 participants (mean age 66.8 y) from the English Longitudinal Study of Ageing, observed in 2018/19 and early (June/July 2020) and later (November/December 2020) in the pandemic. Social isolation is defined using an index including marital status, social contact, and social participation in 2018/19. Using mixed models, we compare changes in well-being, health, health behaviors, financial well-being, and Internet use, between isolated and nonisolated participants. From before to during the pandemic, isolated participants (29%) experienced smaller declines in life satisfaction and quality of life and a smaller increase in loneliness. They showed greater declines in smoking and physical activity and were more likely to remain worried about their future financial situation. They also did not change in their likelihood of regular Internet use, contrasting with nonisolated participants who increased in this regard. The groups followed a similar trend for general health and sleep quality (no change), depression and anxiety (increase), and expectations of future financial difficulties (decrease). Although isolated older adults generally show poorer outcomes than their socially connected counterparts, they were somewhat protected during the pandemic on some fronts. Our findings highlight the need to continually care for isolated older adults but also to be attentive in times of unexpected crises to those experiencing extreme changes related to necessary policy responses.

## Significance

Increased social isolation during the COVID-19 pandemic was worrying, given the known consequences of isolation. However, it is less clear whether older adults who were already isolated before the pandemic reacted differently from those who were socially connected, in terms of well-being during the pandemic. Using a representative sample of adults aged 50+, this study shows similar mental health deteriorations across both groups, but it was the nonisolated group who experienced greater declines in well-being and increase in loneliness. There were also differences in how health behaviors, financial worries, and Internet use changed from before to during the pandemic. Already-isolated adults, despite showing relatively worse outcomes before the pandemic, were somewhat protected, potentially due to fewer changes in their circumstances.

Social isolation has been recognized as a public health problem, given its associations with increased risks of mortality, and an array of physical and mental health conditions (1, 2). This problem was of particular concern during the COVID-19 pandemic, when lockdowns and social distancing restrictions were imposed worldwide (3–5). There was indeed an increase in social isolation in 2020 and 2021, especially among older adults (6, 7), potentially due to their heightened risks of complications and mortality if infected. This was a primary source of stress among older adults (8) and was duly associated with poorer mental health during the pandemic (9, 10).

However, there has been little focus on the experience of those who were already socially isolated before the pandemic. Isolation is the objective deficit in social contacts with others, or the lack or limited extent of integration or connectedness with their social network. This is especially relevant to older adults, due to the circumstances of aging, including relationship losses, retirement, and health and functional declines (11). Objective isolation has been shown to be a more important contributor to mortality than loneliness, or perceived isolation (12, 13). In addition to observing isolation during the pandemic—as an outcome or a predictor of poor well-being—it is important to understand whether older adults who were already isolated before the pandemic were different from those who were socially connected, in terms of their well-being during the pandemic. For instance, there was an overall deterioration in mental health among older adults from before to during the pandemic (14–16), but it is less clear as to whether those who were already isolated experienced a more extreme deterioration than those who were not isolated before the pandemic. Such insight can be useful in targeting social isolation interventions toward a better allocation of resources, besides informing future policies on isolation, quarantine, and social distancing measures.

On the one hand, socially isolated older adults may have fared worse than their nonisolated counterparts. Evidence shows that those living in social isolation are generally more likely to be suffering from physical and mental health conditions than those who are not socially isolated (1). The pandemic and ensuing restrictions may have exacerbated existing health conditions and disparities, due to planned treatments being canceled or postponed, and the vast amount of uncertainty and change brought about by the pandemic. Isolated adults may also have experienced a larger increase in loneliness, with their already limited social activities further reduced. On the other hand, their limited social activities before the pandemic may have meant that when restrictions were imposed, they did not experience a large amount of loss or change in lifestyle. Therefore, they may have experienced smaller changes in terms of their well-being, health, and health behaviors, relative to their nonisolated counterparts who experienced a substantial shock to their social system (17, 18). They may have even been better placed to cope and adapt, potentially via existing routines and arrangements that supported their isolated lives (e.g., delivery of groceries and medication) (19).

To date, longitudinal evidence comparing the experience of those who were already socially isolated before the pandemic with that of those who were not isolated is scarce. Some studies have found that during the pandemic, older adults who were already socially isolated before the pandemic experienced larger increases in depression, anxiety, and loneliness (20–24) and declines in health behaviors, including sleep problems and deteriorations in diet quality and physical activity (25), compared with nonisolated older adults. However, these longitudinal studies on changes over the pandemic by prepandemic isolation status have not necessarily used a) a representative sample, b) a measure of social isolation that captures multiple aspects of older adults’ social lives, and c) an outcome-wide approach to assess different domains including health, well-being, health behaviors, and financial well-being.

The aim of this study is to examine whether older adults who were already socially isolated before the outbreak of the COVID-19 pandemic had different experiences with respect to their well-being during the pandemic, compared with those who were socially connected before the pandemic. We interrogate the English Longitudinal Study of Ageing (ELSA), a nationally representative sample of adults aged 50 y and above in private households in England, with a high response rate (74%) over two COVID-19 waves in June/July 2020 and November/December 2020. We use a composite measure of social isolation that captures multiple aspects of older adults’ social lives, namely partnership status, contact with family and friends, and participation in organizations. This measure has been employed elsewhere, facilitating future evidence comparison and reviews (11, 12). Employing an outcome-wide design (26, 27), we estimate changes in well-being, physical and mental health, loneliness, health behaviors, financial well-being, and Internet use from before (2018/19) to during the pandemic and analyze whether these changes differ by prepandemic isolation status.

The Oxford COVID-19 Government Response Tracker stringency index (ranging 0 to 100) measures the extent of government policy responses pertaining to closure and containment (e.g., cancelation of public events, closure of public transport, restriction on gathering size, stay-at-home requirements) that were in place at the time of data collection (28). The stringency index ranged between 57 and 64 during Wave 1, which covered a period of eased restrictions after the first national lockdown (March 26 to May 12, stringency index 80). This index ranged between 66 and 78 during Wave 2, which covered the entire second national lockdown (November 5 to December 2, stringency index 75 to 78). Given the lower stringency during the June/July 2020 data collection relative to the first weeks of the COVID-19 lockdown, it could be that observed deteriorations in well-being, if any, were less extreme than if data were collected in the earlier weeks. In November/December 2020, stringency was on average higher than that in June/July (closer to levels imposed in the most restrictive period). Observed deteriorations in well-being could therefore be larger during this period, though learning effects could have also led to smaller marginal declines over time.

## Results

### Descriptive Statistics.

Table 1 displays weighted summary statistics for our analytical sample (*n* = 4,636). At baseline (2018/19), 29% were categorized as already socially isolated before the pandemic. Compared with nonisolated participants, isolated participants were more likely to be male and living alone, and to have a limiting, long-term health condition. They also had lower educational attainment, were less likely to be employed and less wealthy, and more likely to live in poorer socioeconomic conditions.

**Table 1.** Distribution of covariates and outcomes by isolation status in 2018/19

	Isolated (N = 1,357)	Nonisolated (N = 3,279)	Total sample (N = 4,636)
Covariates			
Age in 2020	66.908	66.779	66.820
Gender: male	0.504*	0.455	0.470
Ethnicity: white	0.943	0.935	0.938
No. people in household: 0	0.442*	0.124	0.224
No. people in household: 1	0.400*	0.589	0.529
No. people in household: 2	0.092*	0.160	0.139
No. people in household: 3 or more	0.066*	0.127	0.108
Has limiting, long-term health condition	0.393*	0.267	0.307
Living in rural area	0.209*	0.247	0.235
Education: no qualification	0.212*	0.135	0.160
Education: below O-level or other [NVQ1]	0.108	0.113	0.112
Education: O-level [NVQ2]	0.240	0.222	0.228
Education: A-level/higher education below degree [NVQ3]	0.252*	0.286	0.275
Education: Degree [NVQ4 and NVQ5]	0.188*	0.243	0.226
Employment status: Employed	0.308*	0.364	0.346
Employment status: Self-employed	0.087	0.096	0.093
Employment status: Retired	0.465	0.472	0.470
Employment status: Unemployed	0.024*	0.007	0.012
Employment status: Sick or unoccupied	0.116*	0.062	0.079
Wealth: Quintile 1 (bottom, lowest)	0.307*	0.128	0.185
Wealth: Quintile 2	0.211*	0.169	0.182
Wealth: Quintile 3	0.189*	0.221	0.211
Wealth: Quintile 4	0.171*	0.235	0.214
Wealth: Quintile 5 (top, highest)	0.122*	0.248	0.208
Index of multiple deprivation: Quintile 1 (least deprived)	0.170*	0.263	0.234
Index of multiple deprivation: Quintile 2	0.220*	0.252	0.242
Index of multiple deprivation: Quintile 3	0.208	0.212	0.211
Index of multiple deprivation: Quintile 4	0.202*	0.173	0.182
Index of multiple deprivation: Quintile 5 (most deprived)	0.200*	0.100	0.132
Outcomes			
Life satisfaction [0,10]	6.823*	7.657	7.393
CASP-12 quality of life [0,36]	24.418*	27.445	26.490
Poor self-reported health [0,1]	0.331*	0.200	0.241
Depression (4+ of 8 CESD symptoms) [0,1]	0.183*	0.089	0.119
Anxiety [0,10]	2.620*	2.402	2.470
UCLA loneliness [3, 9]	4.563*	3.906	4.114
Currently smoking [0,1]	0.181*	0.075	0.109
Poor sleep quality [0,1]	0.464*	0.423	0.436
Regular physical activity [0,1]	0.582*	0.714	0.673
Poor financial expectations [0,100]	32.018*	28.190	29.402
Worried about future financial situation	n/a	n/a	n/a
Daily Internet use [0,1]	0.692*	0.787	0.757

Notes: Sample means are weighted using longitudinal weights. Asterisks indicate significant differences between isolated and nonisolated participants (*P*s < 0.05).

Our outcomes of interest are described in detail in *SI Appendix*, Table S1, with their pairwise correlations presented in *SI Appendix*, Table S2. In 2018/19 before the pandemic, isolated participants reported lower life satisfaction, quality of life, and physical and mental health, relative to nonisolated participants. They were also lonelier and more likely to smoke and to have poor sleep quality, and less likely to engage in regular physical activity. Compared with nonisolated participants, isolated participants expected higher chances that in the future they will not have enough financial resources to meet their needs and were less likely to use the Internet on a daily basis.

### Main Analysis.

**Fig. 1.**
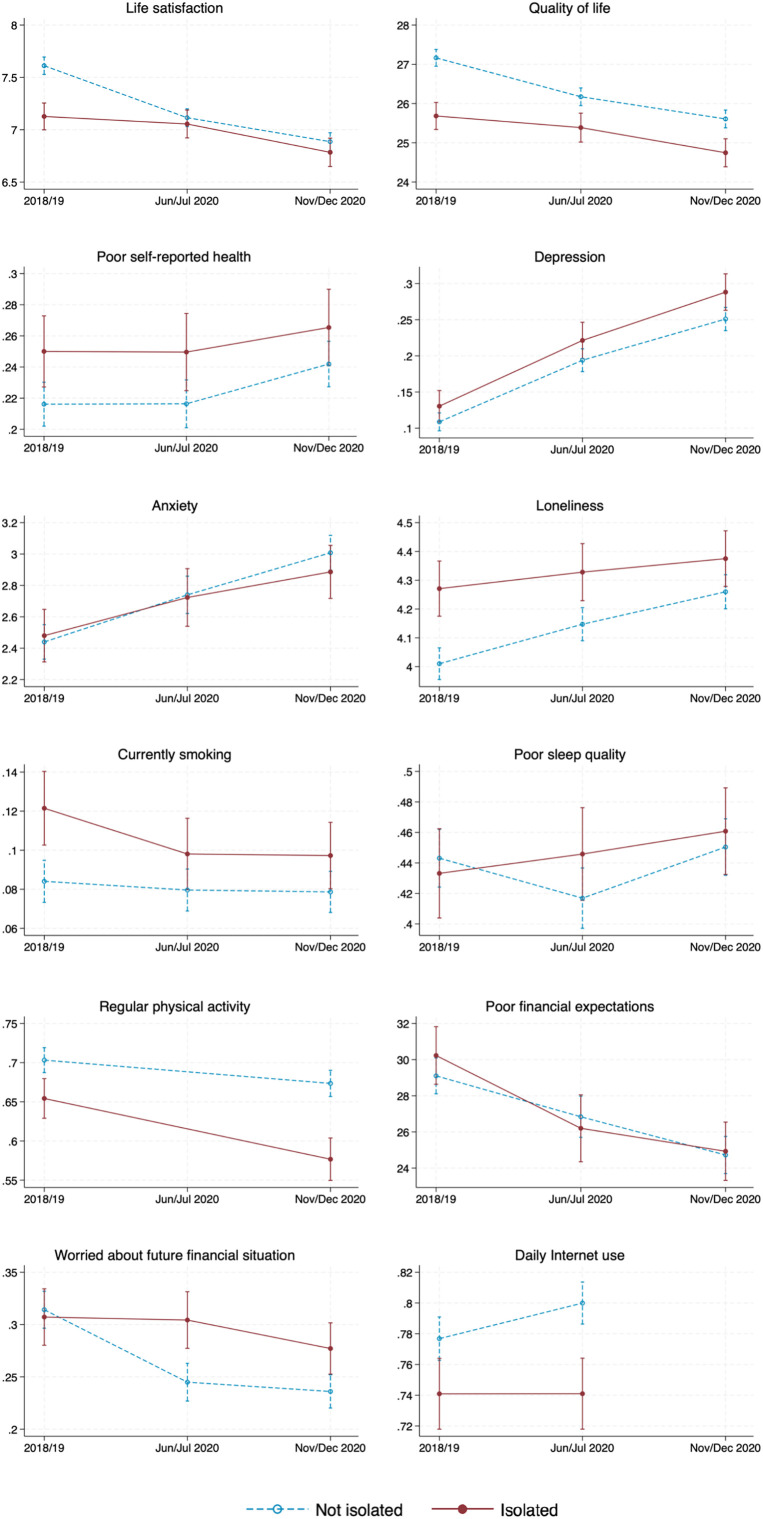
Adjusted estimates (with 95% CI) of the outcomes by wave and prepandemic social isolation status (observed in 2018/19). Estimates for nonisolated participants are in blue (with dashed lines) and estimates for isolated participants are in red (with solid lines). See Table 2 notes for model specification.

For each outcome, we estimate a mixed model that allows for individual differences at baseline and in how the outcome changes between waves. In Table 2, we present the estimated changes between waves for isolated and nonisolated participants, and in Table 3, we estimate the difference between the groups in these changes. To ease discussion, we term the baseline wave (2018/19) as W0 and the first and second COVID-19 waves as W1 (June/July 2020) and W2 (November/December 2020). Fig. 1 plots the adjusted outcome means at each wave by isolation status, estimated from the models in Table 2. Reports of significance are at the 5% level, with 95% CI depicted. All analyses were adjusted for multiple covariates, including age, gender, ethnicity, number of people living in the household, long-term health conditions, education, wealth, and neighborhood deprivation.

**Table 2.** Estimated outcome changes between waves by prepandemic isolation status

	Isolated			Nonisolated		
	W1–W0	W2–W0	W2–W1	W1–W0	W2–W0	W2–W1
Life satisfaction	−0.072	−0.343**	−0.271*	−0.497**	−0.725**	−0.229**
Quality of life	−0.296	−0.938**	−0.641**	−0.992**	−1.557**	−0.566**
Poor self-reported health	−0.000	0.015	0.016	0.000	0.026*	0.026*
Depression	0.091**	0.158**	0.067**	0.085**	0.142**	0.057**
Anxiety	0.243*	0.406**	0.163	0.300**	0.568**	0.268**
Loneliness	0.057	0.104*	0.047	0.137**	0.250**	0.113**
Currently smoking	−0.023*	−0.024**	−0.001	−0.004	−0.005	−0.001
Poor sleep quality^*^	0.013	0.028	0.015	−0.026*	0.007	0.034*
Regular physical activity^†^		−0.078**			−0.030*	
Poor financial expectations	−4.027*	−5.298**	−1.271	−2.263*	−4.379**	−2.116*
Worried about future financial situation^‡^	−0.003	−0.030*	−0.027*	−0.069**	−0.078**	−0.009
Daily Internet use^§^	0.000			0.023**		

Notes: W0 = 2018/19, W1 = June/July 2020, W2 = November/December 2020. Coefficient estimates are from mixed-effects models (weighted using longitudinal weights), where the outcome across all three waves is regressed on isolation status before the pandemic, wave indicators (2018/19 as baseline), and their interaction terms. Models further include covariates measured at baseline, namely age, gender, ethnicity (white vs. otherwise), number of people in the household, disability status, rural status, educational attainment, employment status, wealth quintile, and Index of Multiple Deprivation quintile. *P < 0.05, **P < 0.01.

^*^Outcome in 2018/19: Sleep was restless much of the time during the past week.

^†^Not available at COVID-19 Wave 1.

^‡^Outcome in 2018/19: Reported at least a 50% chance “that at some point in the future you will not have enough financial resources to meet your needs”.

^§^Not available at COVID-19 Wave 2.

**Table 3.** Differences between isolated and nonisolated participants in their estimated outcome changes between waves

	Isolated – nonisolated		
	W1–W0	W2–W0	W2–W1
Life satisfaction	0.425**	0.382**	−0.042
Quality of life	0.695*	0.619**	−0.075
Poor self-reported health	−0.001	−0.010	−0.010
Depression	0.006	0.016	0.010
Anxiety	−0.057	−0.162	−0.105
Loneliness	−0.080	−0.146*	−0.066
Currently smoking	−0.019*	−0.019*	0.000
Poor sleep quality^*^	0.039	0.020	−0.019
Regular physical activity^†^		−0.048*	
Poor financial expectations	−1.764	−0.919	0.845
Worried about future financial situation^‡^	0.066*	0.048*	−0.018
Daily Internet use^§^	−0.023*		

Notes: W0 = 2018/19, W1 = June/July 2020, W2 = November/December 2020. Coefficient estimates are from mixed-effects models (weighted using longitudinal weights), where the outcome across all three waves is regressed on isolation status before the pandemic, wave indicators (2018/19 as baseline), and their interaction terms. Models further include covariates measured at baseline, namely age, gender, ethnicity (white vs. otherwise), number of people in the household, disability status, rural status, educational attainment, employment status, wealth quintile, and Index of Multiple Deprivation quintile. These models are identical to those estimated in Table 2. *P < 0.05, **P < 0.01.

^*^Outcome in 2018/19: Sleep was restless much of the time during the past week.

^†^Not available at COVID-19 Wave 1.

^‡^Outcome in 2018/19: Reported at least a 50% chance “that at some point in the future you will not have enough financial resources to meet your needs”.

^§^Not available at COVID-19 Wave 2.

#### Well-being and health.

For life satisfaction (0 to 10 scale), isolated participants declined (by 0.271) from W1 to W2, with a much smaller change seen from W0 to W1 (0.072). In contrast, nonisolated participants declined steadily from W0 to W2, with the W0 to W1 decline (0.497) being over twice the magnitude of the W1 to W2 decline (0.229). A similar pattern can be observed for quality of life. Therefore, isolated participants appeared to have fared better for life satisfaction and quality of life, particularly from W0 to W1. Fig. 1 duly shows the W0 to W1 declines being larger for nonisolated participants, who began with higher life satisfaction and quality of life at W0, whereas W1 to W2 declines were parallel for the two groups. Notably, life satisfaction ratings in both W1 and W2 were statistically similar between the groups.

There were no significant changes in self-reported health from W0 to W2 for isolated participants, but nonisolated participants were more likely (by 2.6 percentage points) to report having fair/poor health at W2 than at W0. From W0 to W2, both groups increased in the likelihood of depression (15.8 and 14.2 percentage points for isolated and nonisolated participants) and anxiety levels (0.406 and 0.568 on a 0 to 10 scale). However, the groups did not differ with respect to changes in self-reported health, depression, and anxiety. In Fig. 1, changes in these outcomes were largely parallel for the two groups.

The increase in loneliness from W0 to W2 was significantly higher for nonisolated participants than isolated participants (by 0.250 vs. 0.104 on a 3 to 9 scale). Fig. 1 shows that nonisolated participants started with lower loneliness at W0, with a steeper increase from W0 to W2.

#### Health behaviors.

Isolated participants, who were significantly more likely to be smoking at W0 than nonisolated participants, showed a significant decline (by 2.3 percentage points) in smoking from W0 to W1, with no further change from W1 to W2. In contrast, smoking prevalence remained similar throughout W0 to W2 for nonisolated participants.

There were no significant changes in the likelihood of poor sleep quality from W0 to W2 for isolated participants, whereas nonisolated participants first improved from W0 to W1 (by 2.6 percentage points), and then deteriorated back to approximately baseline from W1 to W2 (3.4 percentage points). With respect to the likelihood of regular physical activity, isolated participants showed a larger decline than nonisolated participants from W0 to W2 (7.8 vs. 3.0 percentage points). From Fig. 1, we observe an even larger disparity in physical activity between the groups during the pandemic than before the pandemic.

Given limitations pertaining to alcohol use information collected in W0 and W2, we are not able to provide a meaningful estimate of changes in participants’ alcohol use behavior.

#### Financial well-being.

There were significant declines from W0 to W2 in participants’ expectations of future financial difficulties (i.e., perceived chance that in the future they will not have enough financial resources to meet their needs) and in whether they were worried about their future financial situation. For the former outcome, the W0 to W2 decline was similar between isolated and nonisolated participants (by 5.3 and 4.4 on a 0 to 100 scale). For the latter, nuances can be seen between the waves: There was a prominent W0 to W1 decline (6.9 percentage points) among nonisolated participants but a relatively smaller W1 to W2 decline (2.7 percentage points) among isolated participants. Compared with isolated participants, nonisolated participants were significantly less likely to be worried about their future financial situation by W2 than at W0; though both groups started out with a similar likelihood, as shown in Fig. 1.

#### Internet use.

We consider changes from W0 to W1 in the likelihood of at least daily Internet use (which captures regular use), finding an increase by 2.3 percentage points among nonisolated participants, but no change among isolated participants.

### Sensitivity Checks.

In *SI Appendix*, Fig. S1, we show that we do not use the full representative sample participating in all three waves (*n* = 5,146), due to missingness on ELSA Wave nine baseline information, including on social isolation. Given some differences in those who were excluded from and those included in our final analytical sample (*n* = 4,636) (presented in *SI Appendix*, Table S3), we conduct a sensitivity check of our main results in Tables 2 and 3 with the full sample, filling in missing information via multiple imputation using chained equations. Our model estimates with the imputed data are very similar, as presented in *SI Appendix*, Tables S4 and S5. We nonetheless retain Tables 2 and 3 as our preferred results, following guidance on types of data missingness for which complete case analysis is advised over multiple imputation (29).

We also assess how the main findings change when a different cutoff for prepandemic social isolation is applied. Eight percent of the sample showed scores of three or more of the five criteria for social isolation (cf. 29% scoring two or more), and applying this cutoff resulted in similar findings (presented in *SI Appendix*, Table S6, Column 1). The pattern of differences observed between isolated and nonisolated participants was similar for most outcomes. In contrast to results from using the preferred cutoff, no significant differences were observed for smoking, physical activity, and Internet use, but their estimate sizes remain very similar.

When using “living alone” to define social isolation (*SI Appendix*, Table S6, Column 2), we observe similar findings for life satisfaction, quality of life, self-reported health, depression, anxiety, and sleep quality. However, differences between the groups with respect to changes in loneliness, smoking, physical activity, financial well-being, and Internet use were more muted. That is, there were no clear differences between participants who were already living alone and those not living alone before the pandemic, in how these outcomes changed from before to during the pandemic.

We also consider an individual fixed-effects modeling specification, which estimates within-individual changes in the outcome relative to their own baseline values (W0). With this specification, all time-invariant covariates, such as those associated with selection into the prepandemic social isolation status, are eliminated (this includes the main effect of social isolation, but their interactions with the wave dummies remain). In contrast, the preferred mixed-model specification exploits both within- and between-individual variation, besides allowing us to model individual-specific random variation in the outcome and how the outcome changes across waves (i.e., intercept and wave slope) (30). Estimated differences between the groups in the outcome changes (*SI Appendix*, Table S6, Column 3) are nonetheless similar to those from the preferred specification (Table 3).

## Discussion

This study documents whether older adults who were already socially isolated before the COVID-19 pandemic had different experiences across a wide range of outcomes, from those who were socially connected before the pandemic. To this end, we interrogate the nationally representative ELSA, from before the pandemic in 2018/19, to two timepoints during the pandemic in 2020 (June/July and November/December).

### Well-Being and Health.

Older adults who were already isolated before the pandemic fared better with respect to life satisfaction, quality of life, and loneliness, compared with their nonisolated counterparts. It is likely that the nonisolated group experienced a greater disruption in their habitual routines and rhythms, including missing meaningful social events and gatherings (18), which possibly eroded their sense of meaning and significance (17). In contrast, the already-isolated group may have experienced relatively fewer changes in their daily lives, with their usual routines and arrangements possibly being less prone to disruptions by restrictions during the pandemic (19).

Our sample of older adults experienced declines in health from before to during the pandemic, consistent with evidence elsewhere (14–16). We find that both isolated and nonisolated groups followed a similar pattern of increase in the likelihood of poor self-reported health and depression, and in anxiety levels. This contrasts with past studies which found a larger increase among those who were already socially isolated before the pandemic (20–22). However, these studies differ from ours in the definition of social isolation, where one relied on living alone (21) and the other on perceived social isolation and support (22). Another study was conducted in a substantively different setting, specifically in rural areas within the (single) Shandong province in China in August/September 2020 (20), who were at the time experiencing more extreme restrictions, given the zero-COVID strategy in China. In contrast, we observe a nationally representative sample in England (with only 23.5% from rural areas) in June/July and November/December 2020. Our study adds to this evidence; further, we use data from three time points from before the pandemic until December 2020, whereas these earlier studies (20–22) relied on two time points (one before and one during the pandemic, until September 2020).

### Health Behaviors.

Compared with nonisolated older adults, those already isolated before the pandemic showed larger declines in both regular physical activity and smoking prevalence, from before to during the pandemic. Our physical activity finding is consistent with past findings (25); it may be the case that nonisolated older adults were encouraged by their social network to remain active during the pandemic, such as making use of the allowance (during the most stringent period in W2) to exercise outdoors with household members or one other nonhousehold member.

The observed decline in smoking during the pandemic is consistent with the general trend found in a meta-analysis of 31 studies on smoking after the outbreak of the pandemic (until November 2020, *n* = 269,164 across 24 countries) (31). Considering the increased risk of severe COVID-19 outcomes among older smokers (32), it is possible that the decline in smoking was driven by concerns about their personal COVID-19 susceptibility and severity (33). This observed decline may also be attributed to retail restrictions and stay-at-home requirements during the pandemic, which reduced accessibility to tobacco products.

We did not find clear changes in sleep quality among older adults from before to during the pandemic, consistent with other longitudinal studies (23, 34). This is a positive finding, given concerns that older adults were at increased risk of circadian misalignment and sleep difficulties during the pandemic (35).

### Financial Well-Being.

Contrary to expectations, there was a decline in older adults’ expectations of future financial difficulties (i.e., not having enough financial resources to meet their needs) from before to during the pandemic, with both the isolated and nonisolated groups showing a similar pattern of decline. It could be that their expenditure was substantially reduced due to stay-at-home requirements and closure of public transport and other businesses including retail shops and restaurants, leading to fewer immediate and salient financial concerns, especially if they expected these restrictions to continue.

However, the groups differed with respect to the likelihood of worrying about their future financial situation, where there was a greater decline in worry among nonisolated older adults. This is in line with the literature that financial security is related to social connectedness among older adults (36). That these worries remained high among isolated adults across our observation period may reflect their general inability to rely on instrumental or financial support from social networks (37).

### Internet Use.

Among older adults who were not isolated before the pandemic, the likelihood of regular (daily) Internet use increased from before to during the pandemic. This is consistent with descriptive evidence that nonisolated older adults were more likely to increase their use of the Internet to stay connected during the pandemic (38, 39). In contrast, there was no increase among already-isolated older adults who, on average, had lower educational attainment and wealth and were more likely to live in a deprived neighborhood. This may be reflective of their barriers to digital engagement, including lack of access to good connectivity and equipment, fear and mistrust in technology arising from lack of experience and skills (40), and lack of digital literacy, especially with new online platforms and services necessitated by the pandemic.

### Strengths and Limitations.

A key strength of this study is our use of a nationally representative longitudinal dataset, which achieved a high response rate (74%) during the pandemic. This high response rate was in part due to the use of telephone interviews for participants who were not able to respond online, in contrast with other studies that were restricted to online data collection during the pandemic, which limited participants to those with Internet access and a certain level of technology proficiency (23).

We capture older adults’ experience of social isolation before the pandemic, in contrast with studies on isolation experienced during the pandemic. These can vastly differ: For instance, a US study on 41,443 older women found that nearly half of the sample (43%) changed their frequency of communication with nonhousehold members from before to during the pandemic in 2020 (41). In our own analytical sample, over a quarter (27%) switched their frequency of contact with family or friends between “less than weekly” and “at least weekly”, between 2018/19 and June/July 2020.

Moreover, our outcome-wide approach (i.e., examining changes across an array of conceptually distinct outcomes) provides a fuller picture of already-isolated older adults’ experiences during the pandemic. This approach is insightful given that they fared worse on some outcomes, yet better on others, relative to their socially connected counterparts (26, 27). Our prospective data from both before the pandemic (baseline) and at two timepoints during the pandemic in 2020 also complement studies which examined trajectories observed only during the pandemic. Our ability to adjust for both baseline covariates and outcome values not only mitigates reverse causality concerns (27) but also allows us to relate our findings to participants’ usual experiences (42).

Another strength of this study is our measure of social isolation. We use a representative composite measure that captures multiple aspects of participants’ social lives, namely marital status, contact with others, and participation in organizations. The measure itself has been employed elsewhere (11, 12), easing future work on effect size comparisons and reviews of the social isolation literature. Our contribution is that we are able to apply this to longitudinal analysis comparing the experience of those who were already socially isolated before the pandemic with those who were not isolated. Past studies have explored how living alone may modify outcomes and trajectories during the pandemic, which we explore as a sensitivity check (and find relatively muted results). For example, one study found a larger increase in exercise frequency among older adults living alone than among those living with others (43). Our sensitivity check shows a similar result, but this difference was not significant in our sample. In another study (23), living alone was associated with a larger increase in loneliness, which contrasts with our findings. However, this was a small, nonrepresentative study on a much older cohort (*n* = 137, mean age = 84), which was limited to participants who were able to respond online. Overall, living alone is only one aspect of social isolation, having indeed been shown to be a relatively poor predictor of the adverse health consequences of isolation (44). This could be especially true in an increasingly digital world: For instance, in our sample, 69% of those living alone contacted friends at least weekly via phone, email, or text messaging in 2018/19, vs. only 48% of those socially isolated based on our preferred measure.

With our composite measure, our study additionally complements past findings where individual components of prepandemic social isolation were associated with opposing experiences during the pandemic. For instance, frequent church attendance has been shown to predict a larger increase in loneliness from before to during the pandemic, consistent with our own findings (24). Yet in this same study, network size and daily contact with network members had no predictive power, whereas living with a partner and participation in organizational meetings were instead protective, predicting smaller rises in loneliness. Another study showed that being married, but also living alone, were individually predictive of a greater decline in sleep quality from before to during the pandemic (25). That we capture isolation across multiple aspects renders our findings more summative in nature, which, coupled with past insights, may help inform policies and interventions targeted at socially isolated older adults as well as efforts to improve methods and measures to assess social connectivity (11).

However, this study is not without limitations. We are restricted to observations at two timepoints within the first year of the pandemic (2020), with some inconsistencies in the outcome measurement across waves (particularly for 2 of 12 outcomes considered, i.e., sleep quality and worries about future financial situation, as detailed in *SI Appendix,* Table S1). Interviews were conducted when restrictions were neither the most nor least stringent (*Materials and Methods* section). This inevitably leads to gaps in our findings, compared with, for instance, using data where interviews were conducted monthly or within periods with prolonged restrictions, or using data where there were additional interviews conducted throughout 2021 and 2022. Our hope is that future studies may build on our findings and further probe whether the groups recovered to baseline values in later stages of the pandemic and postpandemic and how this recovery may have differed by isolation status.

Next, our estimates are not causal: Our aim is to describe and compare experiences of isolated and nonisolated older adults with respect to a wide range of outcomes during the pandemic, rather than provide a precise causal effect of the pandemic on these outcomes. Future studies may investigate these aspects, including disentangling specific aspects of the pandemic (e.g., stay-at-home requirements, loss of job/income, economic downturn, COVID-19 infections) that affected each outcome.

In addition, though the outcome-wide approach is advantageous in facilitating comparisons of effect sizes, reducing researcher bias, and increasing research efficiency, our specification does not address changes over time in the exposure and covariates (27). Further, the outcome-wide approach focuses on a single exposure (composite social isolation measure), but future research may build on our findings to examine and potentially compare the roles of the individual social isolation components.

### Conclusion and Implications.

This study reveals that among adults aged 50+ in private households in England, experiences during the COVID-19 pandemic were different between those who were already socially isolated, and those who were socially connected, before the outbreak of the pandemic. From before to during the pandemic in 2020, isolated older adults experienced relatively smaller declines in life satisfaction and quality of life, and a smaller increase in loneliness. They showed greater declines in the likelihood of smoking and engaging in regular physical activity, and were more likely to remain worried about their future financial situation. While nonisolated older adults increased their likelihood of regular Internet use from before to during the pandemic, the isolated group did not show changes in this regard. However, both groups showed similarly no change in self-reported health and sleep quality, similar increases in depression and anxiety, and a similar decrease in their expectations of future financial difficulties.

On balance, this study points toward a need to continually care for older adults living in social isolation, even during periods of restrictions to movement, travel, and gatherings. During the pandemic, previously isolated adults experienced smaller deteriorations in well-being and loneliness and a similar increase in depression and anxiety, but absolute levels remained worse compared with those who were not socially isolated before the pandemic. Additionally, they tended to fare worse on health behaviors, financial well-being, and Internet use outcomes, consistent with prepandemic evidence. We do not expect social isolation levels to remain the same from before to during the pandemic, given the vast changes experienced across among most, if not all, individuals, that directly or indirectly affect their social circumstances. However, that those who were already previously isolated remain worse off than their socially engaged counterparts, suggests that this subgroup may be at a disadvantage even during a time when isolation is “normalized”.

Our findings may be useful to inform public health and other preventive strategies to reduce isolation and related adverse effects, including building age-friendly environments that facilitate in-person interactions (45), designing community-based initiatives that promote social engagement (46), and potentially providing information and communication technologies training to improve digital skills and attitudes (47) and subsidies to remove financial barriers to Internet use (48). On the other hand, in times of unexpected emergencies and crises, it is vital to pay additional attention to older adults experiencing extreme lifestyle changes due to government measures and policy responses.

## Materials and Methods

### Study Design and Participants.

The ELSA is an ongoing biennial panel study representing adults aged 50+ residing in private households in England. The study began in 2002 (Wave 1), with responses from 12,099 individuals from 7,934 households (i.e., representative core members and their partners). This Wave 1 sample was selected from households that previously responded to the Health Survey for England (HSE) in 1998, 1999, and 2001, which is based on postcode address sampling. Refreshment samples of particular age groups (e.g., those newly entering their 50s), again taken from HSE, have been added to the Study in Waves 3, 4, 6, 7, and 9 to ensure it remains representative of those aged 50+. Every 2 y, participants are interviewed on their health, social, psychological, cognitive, and economic circumstances, via computer-assisted personal interviews in their homes and a paper self-completion questionnaire. In addition, every 4 y, nurse visits are conducted to collect biological samples and anthropometric measurements (49). The most recent sweep before the COVID-19 pandemic is Wave 9, collected between June 2018 and July 2019. Cross-sectional Wave 9 weights were calculated to ensure the sample profile matched population proportions with respect to age by sex group and region, based on 2018 mid-year household population estimates by the Office for National Statistics (50).

The ELSA COVID-19 Substudy was conducted twice in 2020 (Wave 1 June 3 to July 26, Wave 2 November 4 to December 20). The Wave 1 (Wave 2) survey was issued to 9,525 (9,150) eligible members, with 7,040 (6,794) interviews completed, achieving a 74% response rate. In both waves, the survey was administered online (83%) or by telephone interview for those who were not able to respond online (17%). As our analysis relied on key information collected before the pandemic, the sample comprised only core members who were also observed in ELSA Wave 9 (51). Of the 8,736 participants in ELSA Wave 9, our final analytical sample comprises 4,636 participants. A sample selection flowchart is presented in *SI Appendix*, Fig. S1.

Ethical approval for ELSA was granted by the South Central—Berkshire Research Ethics Committee (21/SC/0030, 22/03/2021); the COVID-19 Substudy was approved by the University College London Research Ethics Committee. Informed consent was obtained from all participants. The ELSA data can be accessed via the UK Data Service repository, as Study Numbers 5050 (52) and 8688 (53).

### Social Isolation.

Following Steptoe et al. (12), we define participants as being socially isolated before the pandemic (2018/19) if they scored positively on two or more of five criteria: 1) not married or cohabitating; had less than monthly contact (excluding those living in the same household) via meeting up, speaking on the phone, writing or email, or sending or receiving text messages, with 2) children, 3) other family members (e.g., siblings, parents, cousins, grandchildren), and 4) friends; and 5) not participating in organizations, clubs, or societies.

### Outcomes.

We apply an outcome-wide longitudinal design, assessing the effects of a single exposure from a single time point (i.e., social isolation before the pandemic) on multiple subsequent outcomes. This is especially useful when the exposure can affect a range of outcomes—which is likely the case with the pandemic—and may have divergent effects on different outcomes (26, 27). This single-exposure design also mitigates selection bias, assuming the pandemic was an exogenous shock (54).

We assess life satisfaction, quality of life, self-reported health, depression, anxiety, loneliness, smoking, sleep quality, physical activity, expectations of future financial difficulties, worries about future financial situation, and Internet use. This selection of outcomes, though limited to the variables that were measured consistently from before to during the pandemic, is intended to cover a broad range of well-being domains (55). These are defined in detail in *SI Appendix*, Table S1, and their pairwise correlations are presented in *SI Appendix*, Table S2.

### Covariates.

For each outcome, we adjust for the same set of covariates that are causes of the exposure, outcome, or both; but are not neither affected by the exposure nor on the pathway from exposure to outcome (27). All covariates selected are observed prior to the pandemic in 2018/19. Our aim is to capture total changes in well-being from before to during the pandemic, rather than direct effects of the pandemic on well-being (where we would instead adjust for mediators observed during the pandemic).

We consider a range of demographic factors, namely, participants’ age, gender, and ethnicity (i.e., white vs. otherwise), number of people living in their household, whether they had a long-term limiting health condition or disability, and whether they lived in a rural (vs. urban) area. We also include socioeconomic conditions, namely educational attainment, employment status, wealth quintile, and Index of Multiple Deprivation quintile (reflecting the extent of neighborhood deprivation).

### Statistical Analysis.

For each outcome, we estimate a mixed model that accounts for the longitudinal nature of the data as well as individual-level variation in the intercept and wave slope. In other words, the model allows for individual differences in both the outcome and how the outcome changes across waves. We begin with the following specification:

[1] $$\begin{array}{c} Y_{\mathrm{it}}=\alpha +\beta_{1}{\mathrm{iso}}_{i}+\beta_{2}{\mathrm{wave}}_{t}+\beta_{3}{\mathrm{iso}}_{i}\cdot {\mathrm{wave}}_{t}+X_{i}^{\prime }\gamma \\ \;+X_{i}^{\prime }{\mathrm{wave}}_{t}\delta +u_{i,\alpha }+u_{i,\delta }{\mathrm{wave}}_{t}+\varepsilon_{\mathrm{it}}. \end{array}$$

$Y_{\mathrm{it}}$ denotes the outcome of interest for individual *i* at time *t*, where *t* = {0, 1, 2}, representing 2018/19, June/July 2020, and November/December 2020. For outcomes without a corresponding observation in 2018/19, we use a proxy as denoted in the table notes (e.g., Table 2). ${\mathrm{iso}}_{i}$ is a binary variable representing individual *i*’s 2018/19 isolation status (1 if isolated, 0 otherwise), and ${\mathrm{wave}}_{t}$ indicates the COVID-19 wave in which *Y* was observed. ${\mathrm{iso}}_{i}\cdot {\mathrm{wave}}_{t}$, represents their interaction, and its coefficient $\beta_{3}$ provides the difference in *Y* over time between isolated and nonisolated participants.

$X_{i}^{\prime }$ is a vector of time-invariant covariates, and the coefficient vector *γ* represents how differences in these covariates correspond to differences in the overall mean (intercept *α*). As the magnitude of the covariates’ influence on *Y* would be dependent on the specific point in time *t*, the coefficient vector *δ* reflects the multiplicative interactions of the covariates with ${\mathrm{wave}}_{t}$ (56). As for the random components, $u_{i,\alpha }$ represents individual *i*’s deviation from the intercept *α*, $u_{i,\delta }$ represents their deviation in linear growth rate from the overall mean linear growth rate *β*_2_, and *ε_it_* is an individual- and time-specific residual.

We do not expect the outcomes to change in a linear fashion across the timepoints observed. For instance, the largest changes are likely from before the pandemic (*t* = 0) to the first point observed in the pandemic (*t* = 1). Therefore, we replace ${\mathrm{wave}}_{t}$ with dummy variables indicating each wave (omitting *t* = 0 as reference) and include interactions accordingly, which allows us to estimate whether changes in *Y* between waves differ by isolation status. We nevertheless expect that the overall slope is in the same direction from *t* = 0 to *t* = 2, so for simplicity, we retain the linear form of ${\mathrm{wave}}_{t}$ for the random slope section.

[2] $$\begin{array}{c} Y_{\mathrm{it}}=\alpha +\beta_{1}{\mathrm{iso}}_{i}+\beta_{2}{\mathrm{wave}}_{t=1}+\beta_{3}{\mathrm{wave}}_{t=2}+\beta_{4}{\mathrm{iso}}_{i}∙{\mathrm{wave}}_{t=1}+\beta_{5}{\mathrm{iso}}_{i}{\mathrm{wave}}_{t=2}\\ \;+X_{i}^{\prime }\gamma +X_{i}^{\prime }{\mathrm{wave}}_{t}\delta +u_{i,\alpha }+u_{i,\delta }{\mathrm{wave}}_{t}+\varepsilon_{\mathrm{it}}. \end{array}$$

For nonisolated participants, changes in *Y* from 2018/19 to June/July 2020, from 2018/19 to November/December 2020, and from June/July to November/December, are estimated by *β*_2_, *β*_3_, and (*β*_3_ − *β*_2_). For isolated participants, these are estimated by (*β*_2_ + *β*_4_), (*β*_3_ + *β*_5_), and (*β*_3_ + *β*_5_ − *β*_2_ − *β*_4_). Therefore, differences between the groups in these changes are estimated by *β*_4_, *β*_5_, and (*β*_5_ − *β*_4_).

All estimations are conducted using Stata 17.0. Reports of significance are at the 5% level, with 95% CI depicted. For all models, we apply weights designed for longitudinal analysis between the three waves of interest, to minimize bias from differential nonresponse among key subgroups. These weights adjust for ELSA Wave 9 (2018/19) cross-sectional weights, nonresponse to COVID-19 Wave 1 (June/July 2020) contingent on response to ELSA Wave 9, and nonresponse in COVID-19 Wave 2 (November/December 2020) contingent on response in ELSA Wave 9 and COVID-19 Wave 1. Further details regarding these weights are provided in *SI Appendix*, Fig. S1.

## Supplementary Material

Appendix 01 (PDF)

## Data Availability

Anonymized longitudinal survey data have been deposited in UK Data Service as Study Numbers 5050 (52) and 8688 (53).
