# Supplementary material for: Changes in well-being among socially isolated older people during the COVID-19 pandemic: An outcome-wide analysis
Source: Proc Natl Acad Sci U S A. 2024 Apr 22;121(18):e2308697121. doi: 10.1073/pnas.2308697121 (PMC11067447; doi:10.1073/pnas.2308697121)
Supplement: Supplementary file 1 — Appendix 01 (PDF) [file pnas.2308697121.sapp.pdf]

PNAS

Supporting Information for

The impact of the COVID-19 pandemic on socially isolated older people: An outcome-wide analysis.

Claryn S. J. Kung, and Andrew Steptoe

Claryn S. J. Kung
Email: claryn.kung@ucl.ac.uk

This PDF file includes:

Figure S1
Tables S1 to S6
SI References

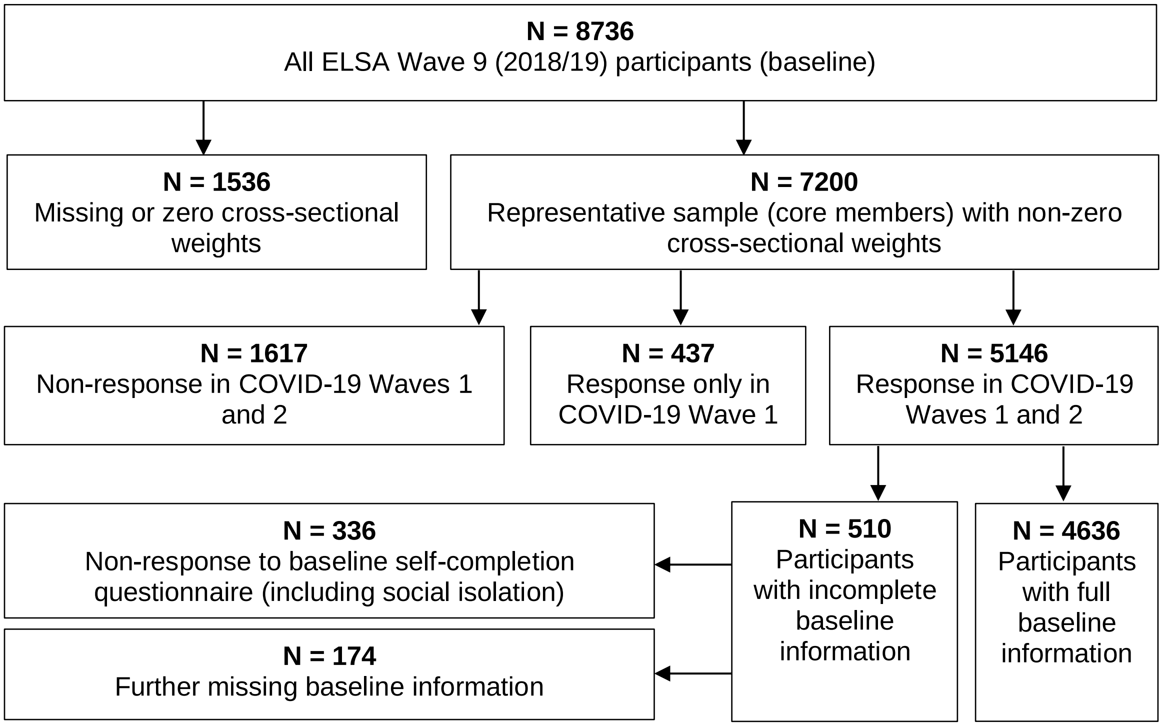

Figure S1. Sample selection flowchart. In ELSA Wave 9 (2018/19), cross-sectional weights applied to 7,200 participants (representative core members) adjusted for differences in the propensity to respond amongst key subgroups. The calculation of these weights also included a scaling factor that ensured the sample represented population proportions with respect to age, based on 2018 mid-year household population estimates by the Office for National Statistics. The remaining 1,536 participants consists of core members' partners (missing cross-sectional weights), or core members who were not living in private households in England (e.g., living in institutions or living outside of England; zero cross-sectional weights) (1).

For the sample of 5,146 participants who were assigned an ELSA Wave 9 cross-sectional weight *and* responded in both COVID-19 Waves 1 and 2, longitudinal weights were calculated. These weights were designed to facilitate analysis of individual-level changes between ELSA Wave 9 and both COVID-19 waves, aimed at minimising bias from differential non-response (2). We apply these weights in all our model estimations.

More specifically, these longitudinal weights accounted for:
(A) Longitudinal weights for COVID-19 Wave 1, which in turn accounted for:
  i. ELSA Wave 9 cross-sectional weight
  ii. Non-response to COVID-19 Wave 1 contingent on response to ELSA Wave 9
(B) Non-response to COVID-19 Wave 2 contingent on response to COVID-19 Wave 1 and ELSA Wave 9

Non-response was modelled using logistic regressions. Taking (A-ii) as an example, the dependent variable was a binary indicator for whether the COVID-19 Wave 1 interview was completed, the predictors were relevant ELSA Wave 9 demographic and behavioural covariates, and the model was weighted using the ELSA Wave 9 cross-sectional weights (predictive covariates were, among others, age group by sex, number of people in the household, educational attainment, employment status, self-reported health, and smoking). The inverse of the predicted probabilities from the model were used to generate the non-response weights, trimmed at the upper 1% tail to exclude extreme

values. The longitudinal weight was then calculated, as a product of the trimmed non-response weight and the ELSA Wave 9 cross-sectional weight (3).

Unfortunately, missing information, primarily from self-completion items from ELSA Wave 9 (which includes the social isolation measure) reduces the sample from 5,146 to 4,636 participants. In Table S3, we present the distribution of covariates at baseline (ELSA Wave 9, 2018/19) between this final working sample and the 510 excluded participants.

Table S1. Outcome definitions.

Outcome	Definition
Life satisfaction [0,10]	Across all waves, as part of the four-item ONS (Office for National Statistics) personal wellbeing questions, participants were asked, "On a scale of 0 to 10, where 0 is "not at all" and 10 is "very", how satisfied are you with your life nowadays?".
CASP-12 quality of life [0,36]	Across all waves, participants were asked, "Here is a list of statements that people have used to describe their lives or how they feel. How often do you feel like this?".<br>1. My age prevents me from doing the things I would like to do<br>2. I feel that what happens to me is out of my control<br>3. I feel left out of things<br>4. I can do the things I want to do<br>5. I feel that I can please myself what I do<br>6. Shortage of money stops me from doing things I want to do<br>7. I look forward to each day<br>8. I feel that my life has meaning<br>9. I enjoy the things that I do<br>10. I feel full of energy these days<br>11. I feel that life is full of opportunities<br>12. I feel that the future looks good for me<br>These 12 statements were taken from the 19-item CASP (Control, Autonomy, Self-realisation, and Pleasure) scale (4). Participants responded "often" (0), "sometimes" (1), "not often" (2), or "never" (3) to each statement. We sum these 12 item responses to obtain a score between 0 and 36, reverse coding as necessary, such that higher scores reflect a higher quality of life.
Poor self-reported health [0,1]	In 2018/19 (ELSA Wave 9), participants were asked, "Would you say your health is…", where response options were "excellent", "very good", "good", "fair", and "poor". In both COVID-19 waves, participants were asked, "In the past month would you say your health was...", with the same response options. We dichotomise this outcome to indicate "fair" or "poor" health (1), vs. "excellent", "very good", or "good" health (0).
Depression (4+ of 8 CESD symptoms) [0,1]	Across all waves, participants were asked whether "much of the time during the past week", "you felt depressed", "you felt that everything you did was an effort", "your sleep was restless", "you were happy", "you felt lonely", "you enjoyed life", "you felt sad", and "you could not get going". These eight items, to each of which they could respond "yes" or "no", were from the Center for Epidemiologic Studies Depression Scale (CES-D) (5). We code every positive response as 1 (reverse-coding for "you were happy" and "you enjoyed life") and use a binary variable to indicate four or more positive responses (1), following other studies (e.g., 6), vs. fewer than four positive responses (0).
Anxiety (10-21 on GAD-7 scale) [0,1]	Across all waves, as part of the four-item ONS (Office for National Statistics) personal wellbeing questions, participants were asked, "On a scale of 0 to 10, where 0 is "not at all" and 10 is "very", how anxious, overall, did you feel yesterday?".<br><br>In both COVID-19 waves, participants were also administered the GAD-7 scale (described below). However, this was not administered in 2018/19 (ELSA Wave 9). When we dichotomise our preferred measure to indicate scores of between 7 and 10 (1), vs. scores between 0 and 6 (0), and observe changes between this

	dichotomised measure in 2018/19 and the GAD-7 score in the two COVID-19 waves, we arrive at very similar conclusions.<br><br>For the GAD-7 (Generalized Anxiety Disorder) scale (4), participants were asked, “Over the last two weeks, how often have you been bothered by any of the following problems?”.<br>1. Feeling nervous, anxious or on edge<br>2. Not being able to stop or control worrying<br>3. Worrying too much about different things<br>4. Trouble relaxing<br>5. Being so restless that it is hard to sit still<br>6. Becoming easily annoyed or irritable<br>7. Feeling afraid as if something awful might happen<br>Participants responded “not at all” (0), “several days” (1), “more than half the days” (2), or “nearly every day” (3) to each statement. We sum these seven item responses to obtain a score between 0 and 21, such that higher scores reflect higher levels of anxiety, and use a binary variable to indicate scores between 10 and 21 (1) vs. scores between 0 and 9 (0), which reflect moderate to severe anxiety levels (7).
UCLA loneliness [3,9]	Across all waves, participants were administered the three-item Revised UCLA (University of California, Los Angeles) Loneliness Scale (8), consisting of “How often do you feel you lack companionship?”, “How often do you feel left out?”, and “How often do you feel isolated from others?”. Response options for each item were “hardly ever or never” (1), “sometimes” (2), and “often” (3). We sum these three item responses to obtain a score between three and nine, such that higher scores reflect higher levels of loneliness.
Currently smoking [0,1]	In 2018/19 (ELSA Wave 9), participants were asked, “Have you ever smoked cigarettes?” and “Do you smoke cigarettes at all nowadays?”, where response options to each were “yes” or “no”. We mark participants responding “no” to both questions as not currently smoking (0), and participants responding “yes” to the second question as currently smoking (1). In both COVID-19 waves, participants were asked, “Do you currently smoke?”, where response options were “yes” (1) or “no” (0).
Poor sleep quality [0,1]	In 2018/19 (ELSA Wave 9), participants were asked, “Much of the time during the past week, your sleep was restless?”, where response options were “yes” (1) or “no” (0). This question was taken from the Center for Epidemiologic Studies Depression Scale (CES-D; Radloff 1977). In a sensitivity check we also examine a related item, “Did you feel well-rested yesterday morning (that is you slept well the night before)?”, where response options were “yes” (0) or “no” (1), finding qualitatively identical conclusions. We retain the former item as our preferred 2018/19 measure of poor sleep quality, given the longer time frame considered. In both COVID-19 waves, participants were asked, “In the past month would you say the quality of your sleep was...”, where response options were “excellent”, “very good”, “good”, “fair”, and “poor”. We dichotomise this outcome to indicate “fair” or “poor” sleep quality (1), vs. “excellent”, “very good”, or “good” quality (0).
Regular physical activity [0,1]	In 2018/19 (ELSA Wave 9), participants were asked, “Do you take part in sports or activities that are vigorous…” and “Do you take part in sports or activities that are moderately energetic…”, where response options to each were “more than once a week”, “once a week”, “one to three times a month”, and “hardly ever, or never”. In November/December 2020 (COVID-19 Wave 2), participants were asked, “How often, if ever, do you take part in sports or activities that are vigorous?” and “How often, if ever, do you take part in sports or activities that are

	moderately energetic?", with the same response options. We dichotomise this outcome to indicate participation in vigorous or moderately energetic activities more than once a week (1), vs. lower frequencies (0). These physical activity questions were not administered in June/July 2020 (COVID-19 Wave 1).
Poor financial expectations [0,100]	Across all waves, participants were asked, "On a scale of 0 to 100, where 0 means there is absolutely no chance it will happen and 100 means it is certain to happen, what are the chances that at some point in the future you will not have enough financial resources to meet your needs?".
Worried about future financial situation	In both COVID-19 waves, participants were asked, "How worried, if at all, are you about your future financial situation?", where response options were "not at all worried", "not very worried", "somewhat worried", "very worried", and "extremely worried". We dichotomise this outcome to indicate "somewhat worried", "very worried", or "extremely worried" (1), vs. "not at all worried" or "not very worried" (0).<br><br>This question was not administered in 2018/19 (ELSA Wave 9), so we use the "poor financial expectations" measure from this wave. We dichotomise this outcome to indicate reports of at least a 50% chance "that at some point in the future you will not have enough financial resources to meet your needs".
Daily Internet use [0,1]	We are also interested in participants' Internet use behaviour, which has been shown to be helpful to mitigate isolation concerns (9).<br><br>In 2018/19 (ELSA Wave 9), participants were asked, "On average, how often do you use the Internet or email?", where response options were "every day, or almost every day", "at least once a week (but not every day)", "at least once a month (but not every week)", "at least once every 3 months", "less than every 3 months", and "never". In June/July 2020 (COVID-19 Wave 1), participants were asked, "Since the coronavirus outbreak, on average, how often did you use the internet or email?", where response options were "more than once a day", "every day, or almost every day", "at least once a week (but not every day)", "at least once a month (but not every week)", "less than monthly", and "never". We dichotomise this outcome to indicate "more than once a day" or "every day, or almost every day" (1), vs. lower frequencies (0). This Internet use question was not administered in November/December 2020 (COVID-19 Wave 2).

Notes: In ELSA Wave 9, items related to self-reported health, smoking, physical activity, depression, sleep quality, expectations of future financial difficulties, and worries about future financial situation, were administered (in this order) via computer-assisted personal interviews, whereas Internet use, loneliness, quality of life, anxiety, and life satisfaction were asked (in this order) in the paper self-completion questionnaire. Participants were not given specific instructions to complete the questionnaire before or after the personal interview; some were also given the questionnaire ahead of the interview (10). Note that for social isolation, marital status was from one of the earliest modules administered in the Wave 9 computer-assisted personal interview (individual demographics). The other four social isolation components were asked in the Wave 9 self-completion questionnaire, in the order of participation in organisations (prior to Internet use) and contact with children, other family members and friends (after quality of life, prior to anxiety). All covariates were derived from the Wave 9 computer-assisted personal interview, including from modules pertaining to individual demographics, work and pensions, and income and assets.

In COVID-19 Waves 1 and 2, participants were first invited to take part in the study online (computer-assisted web interview). Only after they were offered enough time and opportunities to participate online, they were contacted by an interviewer and invited to complete a computer-assisted telephone interview. In these COVID-19 Waves, the order of the outcome items was

depression, anxiety, life satisfaction, quality of life, worries about future financial situation, physical activity, smoking, self-reported health, sleep quality, Internet use, loneliness, and expectations of future financial difficulties.

Table S2. Pairwise outcome correlation matrix.

	Outcome (W0)	1	2	3	4	5	6	7	8	9	10	11	12
1	Life satisfaction	1											
2	Quality of life	0.697*	1										
3	Poor self-reported health	-0.298*	-0.426*	1									
4	Depression	-0.425*	-0.422*	0.278*	1								
5	Anxiety	-0.385*	-0.416*	0.164*	0.307*	1							
6	Loneliness	-0.527*	-0.565*	0.217*	0.424*	0.330*	1						
7	Currently smoking	-0.101*	-0.125*	0.099*	0.074*	0.032*	0.066*	1					
8	Poor sleep quality [a]	-0.229*	-0.262*	0.184*	0.279*	0.184*	0.198*	0.035*	1				
9	Regular physical activity [b]	0.137*	0.251*	-0.302*	-0.153*	-0.063*	-0.122*	-0.068*	-0.125*	1			
10	Poor financial expectations	-0.228*	-0.283*	0.130*	0.156*	0.145*	0.157*	0.072*	0.133*	-0.056*	1		
11	Worried about future financial situation [c]												
12	Daily Internet use [d]	0.084*	0.164*	-0.146*	-0.086*	0.009	-0.074*	-0.065*	-0.071*	0.134*	-0.033*		1

	Outcome (W1)	1	2	3	4	5	6	7	8	9	10	11	12
1	Life satisfaction	1											
2	Quality of life	0.594*	1										
3	Poor self-reported health	-0.282*	-0.473*	1									
4	Depression	-0.449*	-0.546*	0.324*	1								
5	Anxiety	-0.257*	-0.376*	0.201*	0.357*	1							
6	Loneliness	-0.468*	-0.576*	0.255*	0.494*	0.292*	1						
7	Currently smoking	-0.035*	-0.070*	0.093*	0.042*	0.008	0.051*	1					
8	Poor sleep quality [a]	-0.310*	-0.376*	0.325*	0.327*	0.253*	0.267*	0.057*	1				
9	Regular physical activity [b]												
10	Poor financial expectations	-0.164*	-0.211*	0.083*	0.116*	0.129*	0.169*	0.049*	0.133*		1		

11	Worried about future financial situation [c]	-0.236*	-0.364*	0.191*	0.203*	0.179*	0.248*	0.083*	0.200*		0.372*	1	
12	Daily Internet use [d]	-0.022	0.116*	-0.154*	-0.053*	-0.025	-0.027	-0.077*	-0.018		0.090*	-0.003	1

	Outcome (W2)	1	2	3	4	5	6	7	8	9	10	11	12
1	Life satisfaction	1											
2	Quality of life	0.606*	1										
3	Poor self-reported health	-0.285*	-0.470*	1									
4	Depression	-0.492*	-0.605*	0.335*	1								
5	Anxiety	-0.293*	-0.415*	0.185*	0.408*	1							
6	Loneliness	-0.482*	-0.588*	0.260*	0.534*	0.341*	1						
7	Currently smoking	-0.040*	-0.081*	0.080*	0.055*	0.002	0.074*	1					
8	Poor sleep quality [a]	-0.311*	-0.398*	0.336*	0.352*	0.248*	0.263*	0.028	1				
9	Regular physical activity [b]	0.090*	0.260*	-0.272*	-0.138*	-0.068*	-0.118*	-0.082*	-0.099*	1			
10	Poor financial expectations	-0.157*	-0.222*	0.069*	0.143*	0.149*	0.159*	0.015	0.134*	-0.022	1		
11	Worried about future financial situation [c]	-0.238*	-0.366*	0.165*	0.259*	0.192*	0.270*	0.103*	0.219*	-0.066*	0.357*	1	
12	Daily Internet use [d]												

Notes: n = 4,636. W0 = 2018/19, W1 = June/July 2020, W2 = November/December 2020.
*p<0.05.
[a] Outcome in 2018/19: Sleep was restless much of the time during the past week. Outcome in COVID-19 Waves: Quality of sleep in the past month was fair or poor (vs. excellent, very good, or good).
[b] Not available at COVID-19 Wave 1.
[c] Outcome in 2018/19: Reported at least a 50% chance “that at some point in the future you will not have enough financial resources to meet your needs”. Outcome in COVID-19 Waves: Somewhat, very, or extremely (vs. not at all or not very) worried about future financial situation.
[d] Not available at COVID-19 Wave 2.

Table S3. Distribution of covariates by sample inclusion after weighting.

	Final analytical sample (N=4636)	Sample not included (N=510)	Full representative sample at COVID-19 Wave 2 (N=5146)
Age in 2020	66.820	65.778	66.678
Gender: male	0.470	0.477	0.471
Ethnicity: white	0.938*	0.865	0.928
No. people in household: 0	0.224	0.214	0.223
No. people in household: 1	0.529*	0.453	0.519
No. people in household: 2	0.139	0.186	0.145
No. people in household: 3 or more	0.108	0.147	0.113
Has limiting, long-term health condition	0.307*	0.368	0.315
Living in rural area	0.235	0.206	0.231
Education: no qualification	0.160*	0.232	0.169
Education: below O-level or other [NVQ1]	0.112*	0.185	0.122
Education: O-level [NVQ2]	0.228	0.207	0.225
Education: A-level/higher education below degree [NVQ3]	0.275*	0.225	0.268
Education: Degree [NVQ4 and NVQ5]	0.226*	0.152	0.216
Employment status: Employed	0.346	0.405	0.354
Employment status: Self-employed	0.093	0.079	0.091
Employment status: Retired	0.470*	0.367	0.456
Employment status: Unemployed	0.012	0.013	0.012
Employment status: Sick or unoccupied	0.079*	0.135	0.087
Wealth: Quintile 1 (bottom, lowest)	0.185*	0.304	0.199
Wealth: Quintile 2	0.182	0.192	0.183
Wealth: Quintile 3	0.211	0.191	0.208
Wealth: Quintile 4	0.214*	0.152	0.207
Wealth: Quintile 5 (top, highest)	0.208*	0.160	0.202
Index of Multiple Deprivation: Quintile 1 (least deprived)	0.234	0.260	0.237
Index of Multiple Deprivation: Quintile 2	0.242*	0.166	0.231
Index of Multiple Deprivation: Quintile 3	0.211	0.172	0.206
Index of Multiple Deprivation: Quintile 4	0.182	0.219	0.187
Index of Multiple Deprivation: Quintile 5 (most deprived)	0.132	0.184	0.139

Notes: Sample means are weighted using longitudinal weights. Asterisks indicate significant differences between included and excluded participants (*p*s<0.05).

Figure S1 illustrates how missing information, primarily from self-completion items from ELSA Wave 9 (which includes the social isolation measure) reduces the sample from 5,146 to 4,636 participants. In Table S3 above, we present the distribution of covariates at baseline (ELSA Wave 9, 2018/19) between this final working sample and the 510 excluded participants. There are differences between those included in and those excluded from our analytical sample, by educational attainment and wealth, among others.

We therefore conduct a sensitivity check of our main results in Tables 2 and 3, using the entire sample of 5,146 participants, and filling in missing information with multiple imputation using chained equations. We present the resulting estimates in Tables S4 and S5.

Table S4. Estimated changes between waves by pre-pandemic isolation status, using 20 multiply imputed datasets.

	Isolated			Non-isolated		
	W1-W0	W2-W0	W2-W1	W1-W0	W2-W0	W2-W1
Life satisfaction	-0.100	-0.320**	-0.220*	-0.499**	-0.687**	-0.188**
Quality of life	-0.372*	-0.944**	-0.572**	-1.001**	-1.538**	-0.536**
Poor self-reported health	-0.001	0.013	0.014	0.001	0.024*	0.023*
Depression	0.094**	0.164**	0.070**	0.085**	0.144**	0.059**
Anxiety	0.245*	0.452**	0.207	0.309**	0.555**	0.246**
Loneliness	0.063	0.096*	0.033	0.142**	0.243**	0.100**
Currently smoking	-0.024*	-0.026**	-0.002	-0.004	-0.006	-0.002
Poor sleep quality [a]	0.007	0.028	0.021	-0.023	0.010	0.033*
Regular physical activity [b]		-0.079**			-0.027*	
Poor financial expectations	-4.894**	-6.024**	-1.130	-2.789**	-4.757**	-1.968*
Worried about future financial situation [c]	-0.009	-0.038*	-0.029*	-0.073**	-0.083**	-0.010
Daily Internet use [d]	-0.001			0.025**		

Notes: W0 = 2018/19, W1 = June/July 2020, W2 = November/December 2020. Coefficient estimates are from mixed-effects models (weighted using longitudinal weights), where the outcome across all three waves is regressed on isolation status before the pandemic, wave indicators (2018/19 as baseline), and their interaction terms. Models further include covariates measured at baseline, namely age, gender, ethnicity (white vs. otherwise), number of people in the household, disability status, rural status, educational attainment, employment status, wealth quintile, and Index of Multiple Deprivation quintile. N = 5,146, where missing values on multiple variables are imputed using chained equations (20 imputations for each outcome).

*p<0.05, **p<0.01.

[a] Outcome in 2018/19: Sleep was restless much of the time during the past week.

[b] Not available at COVID-19 Wave 1.

[c] Outcome in 2018/19: Reported at least a 50% chance “that at some point in the future you will not have enough financial resources to meet your needs”.

[d] Not available at COVID-19 Wave 2.

Table S5. Differences between isolated and non-isolated participants in their estimated outcome changes between waves, using 20 multiply imputed datasets.

	Isolated – Non-isolated		
	W1-W0	W2-W0	W2-W1
Life satisfaction	0.399**	0.367**	-0.032
Quality of life	0.630*	0.594**	-0.036
Poor self-reported health	-0.002	-0.011	-0.009
Depression	0.009	0.020	0.011
Anxiety	-0.064	-0.103	-0.039
Loneliness	-0.080	-0.147*	-0.068
Currently smoking	-0.020*	-0.020*	0.000
Poor sleep quality [a]	0.030	0.018	-0.012
Regular physical activity [b]		-0.051*	
Poor financial expectations	-2.105	-1.267	0.838
Worried about future financial situation [c]	0.064*	0.045*	-0.019
Daily Internet use [d]	-0.026*		

Notes: W0 = 2018/19, W1 = June/July 2020, W2 = November/December 2020. Coefficient estimates are from mixed-effects models (weighted using longitudinal weights), where the outcome across all three waves is regressed on isolation status before the pandemic, wave indicators (2018/19 as baseline), and their interaction terms. Models further include covariates measured at baseline, namely age, gender, ethnicity (white vs. otherwise), number of people in the household, disability status, rural status, educational attainment, employment status, wealth quintile, and Index of Multiple Deprivation quintile. N = 5,146, where missing values on multiple variables are imputed using chained equations (20 imputations for each outcome). These models are identical to those estimated in Table S4.
*p<0.05, **p<0.01.
[a] Outcome in 2018/19: Sleep was restless much of the time during the past week.
[b] Not available at COVID-19 Wave 1.
[c] Outcome in 2018/19: Reported at least a 50% chance "that at some point in the future you will not have enough financial resources to meet your needs".
[d] Not available at COVID-19 Wave 2.

Given differences between excluded and included participants, including in educational attainment and wealth (see Table S3), we conduct a sensitivity check of our main results in Tables 2 and 3 using the entire sample of 5,146 participants. We fill in missing values in multiple variables iteratively, by using chained equations. This method is a sequence of univariate imputation methods with fully conditional specification of prediction equations, which accommodates arbitrary missing-value patters and sampling weights, allowing us to apply the longitudinal weights described above. For each regression model we create 20 imputations (each using 10 burn-in iterations before imputed values are drawn), to account for possible dependence of the estimated model parameters on imputed data. Then, Rubin's combination rules are used to obtain estimates for our regression models from the multiply imputed data.

Results are shown in Tables S4 and S5 above, revealing very little difference in estimates from the main results presented in Tables 2 and 3. Given that most of our missingness is from the exposure (i.e., social isolation in 2018/19), and that the categorical non-response to the self-completion questionnaire is unlikely to be completely explained by observables (i.e., missing not at random), we retain the complete case analysis with 4,636 participants. With data such as ours, complete case analysis has been found to be appropriate and valid, whereas multiple imputation may provide biased results, with little gains in efficiency (11).

Table S6. Estimated changes between waves by pre-pandemic isolation status: Sensitivity checks.

Difference by isolation status (Isolated – Non-isolated)	(1) Using a cut-off of ≥3 to define social isolation			(2) Using living alone as isolation measure			(3) Using fixed-effects models		
	W1-W0	W2-W0	W2-W1	W1-W0	W2-W0	W2-W1	W1-W0	W2-W0	W2-W1
Life satisfaction	0.487**	0.449**	-0.038	0.458**	0.344**	-0.114	0.402**	0.340*	-0.062
Quality of life	0.839*	1.080**	0.241	0.216	0.346	0.130	0.697*	0.613*	-0.084
Poor self-reported health	-0.003	0.004	0.007	-0.009	-0.008	0.001	0.003	-0.002	-0.006
Depression	-0.016	0.033	0.049	-0.009	-0.003	0.006	0.014	0.031	0.017
Anxiety	-0.016	-0.125	-0.109	-0.344*	-0.100	0.243	-0.050	-0.145	-0.095
Loneliness	-0.171	-0.283*	-0.112	0.091	0.067	-0.024	-0.071	-0.125*	-0.054
Currently smoking	-0.023	-0.027	-0.004	-0.002	-0.009	-0.007	-0.025*	-0.030*	-0.006
Poor sleep quality [a]	0.025	0.012	-0.013	-0.019	-0.036	-0.018	0.044	0.032	-0.013
Regular physical activity [b]		-0.036			0.016			-0.053*	
Poor financial expectations	0.685	-3.467	-4.151	-2.110	-2.943*	-0.834	-2.021	-1.394	0.627
Worried about future financial situation [c]	0.058	0.059*	0.001	0.013	-0.011	-0.024	0.072*	0.058*	-0.013
Daily Internet use [d]	-0.026			0.008				-0.034*	

Notes: W0 = 2018/19, W1 = June/July 2020, W2 = November/December 2020. Column 1 in this table follows the same estimations as Table 3 but redefines pre-pandemic social isolation as scoring positively on three or more of five criteria (cf. on two or more of five criteria, in the preferred definition). Column 2 in this table follows the same estimations as Table 3 but replaces the preferred isolation measure with ‘living alone’ before and during the pandemic, and excluding ‘number of people in the household’ (strongly related to the isolation measure) from the model. Column 3 in this table follows the same estimations as Table 3 but uses an individual-level fixed-effects specification; this drops all time-invariant variables from the model, retaining only wave indicators and their interactions with isolation status.

*p<0.05, **p<0.01.

[a] Outcome in 2018/19: Sleep was restless much of the time during the past week.

[b] Not available at COVID-19 Wave 1.

[c] Outcome in 2018/19: Reported at least a 50% chance “that at some point in the future you will not have enough financial resources to meet your needs”.

[d] Not available at COVID-19 Wave 2.

SI References

1. NatCen Social Research, *User guide to the main interview datasets: Waves 1 to 9* (NatCen Social Research, 2020) https://www.ucl.ac.uk/epidemiology-health-care/sites/epidemiology-health-care/files/5050_elsa_waves_1-9_interviewer_data_user_guide_v1.pdf.
2. NatCen Social Research, *User guide for ELSA COVID-19 Substudy: Waves 1 and 2* (NatCen Social Research, 2021) http://doc.ukdataservice.ac.uk/doc/8688/mrdoc/pdf/8688_elsa_covid-19_substudy_user_guide_final.pdf.
3. G. Addario, P. Dangerfield, D. Hussey, B. Pacchiotti, M. Wood, *Adapting fieldwork during the COVID-19 outbreak: A methodological overview of the ELSA COVID-19 Substudy (wave 1)* (NatCen Social Research, 2020) https://11a183d6-a312-4f71-829a-79ff4e6fc618.filesusr.com/ugd/540eba_b8627be63ccb4ecd9bdc41b1fd1b7d52.pdf.
4. M. Hyde, R. D. Wiggins, P. Higgs, D. B. Blane, A measure of quality of life in early old age: The theory, development and properties of a needs satisfaction model (CASP-19). *Aging Ment. Health* **7**, 186-194 (2003).
5. L. S. Radloff, The CES-D scale: A self-report depression scale for research in the general population. *Appl. Psychol. Meas.* **1**, 385-401 (1977).
6. A. Steptoe, D. Fancourt, Leading a meaningful life at older ages and its relationship with social engagement, prosperity, health, biology, and time use. *Proc. Natl. Acad. Sci. U.S.A.* **116**, 1207-1212 (2019).
7. R. L. Spitzer, K. Kroenke, J. B. W. Williams, B. Löwe, A brief measure for assessing generalized anxiety disorder: The GAD-7. *Arch. Intern. Med.* **166**, 1092-1097 (2006).
8. M. E. Hughes, L. J. Waite, L. C. Hawkley, J. T. Cacioppo, A short scale for measuring loneliness in large surveys: Results from two population-based studies. *Res. Aging* **26**, 655-672 (2004).
9. S. Peng, A. R. Roth, Social isolation and loneliness before and during the COVID-19 pandemic: A longitudinal study of U.S. adults older than 50. *J. Gerontol. B Psychol. Sci. Soc. Sci.* **77**, e185-e190 (2021).
10. B. Pacchiotti, D. Hussey, G. Bennett, *The dynamics of ageing: The 2018/2019 English Longitudinal Study of Ageing (Wave 9) technical report* (NatCen Social Research, 2021) https://www.ucl.ac.uk/epidemiology-health-care/sites/epidemiology_health_care/files/elsa_w9_technical_report_final.pdf.
11. R. A. Hughes, J. Heron, J. A. C. Sterne, K. Tilling, Accounting for missing data in statistical analyses: Multiple imputation is not always the answer. *Int. J. Epidemiol.* **48**, 1294-1304 (2019).
